# Association of Changes in Relevant Indicators With Cardiovascular Disease and Osteoporosis in Perimenopausal and Postmenopausal Women

**DOI:** 10.1002/fsn3.4512

**Published:** 2024-11-06

**Authors:** Xiaoyan Luo, Jun Zhang, Yichuan Guo, Liangzhi Xu

**Affiliations:** ^1^ Department of Obstetrics and Gynecology, West China Second University Hospital Sichuan University Chengdu China; ^2^ Key Laboratory of Birth Defects and Related Diseases of Women and Children Ministry of Education, Sichuan University Chengdu China; ^3^ Reproductive Endocrinology and Regulation Laboratory West China Second University Hospital Sichuan University Chengdu China; ^4^ Department of Obstetrics Xishuangbanna Dai Autonomous Prefecture People's Hospital Jinghong China

**Keywords:** cardiovascular disease, estradiol, osteoporosis, perimenopausal, postmenopausal

## Abstract

The essence of menopause is ovarian failure, decreased estrogen volatility, and deficiency leading to multiple related symptoms and an increased risk of metabolic disease in women, such as cardiovascular disease and osteoporosis. This study screened 773 eligible postmenopausal and perimenopausal women from an initial pool of 1187 participants, and various physiological and biochemical indices were measured and analyzed to assess differences across three age groups (40–44 years, 45–49 years, 50–54). We found no significant difference in the rate of cardiovascular disease between postmenopausal and perimenopausal women, while the rate of osteoporosis was higher in postmenopausal women compared to perimenopausal women. The disease of osteoporosis in postmenopausal women was associated with age (*p* < 0.05). We also found that postmenopausal women and perimenopausal women had significant effects on follicle‐stimulating hormone (FSH), luteinizing hormone (LH), estradiol (E_2_), total cholesterol (TC), lumbar spine BMD (T_1_), femoral neck BMD, The bone density (T_2_) of the right femur was significantly affected. There are significant differences in FSH, LH, E_2_, TC, low‐density lipoprotein (LDL), L2–L4, T_1_, Neck of femur decrease, and T_2_ in women of different ages. Furthermore, the correlation analysis between age and E_2_ and metabolic indicators showed that age has a greater impact on the risk of postmenopausal and perimenopausal females. This study can help further understand the mechanisms of cardiovascular disease and osteoporosis in perimenopausal and menopausal women.

## Graphical abstract

773 eligible postmenopausal and perimenopausal women were analyzed in three age groups (40–44, 45–49, 50–54 years). Cardiovascular disease rates showed no significant differences, but osteoporosis was more common in postmenopausal women and linked to age (*p* < 0.05). FSH, LH, E2, TC, and bone density (T1, T2) were significantly affected across groups.

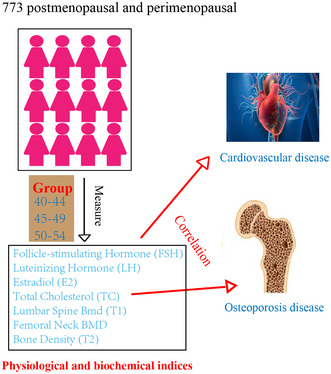

## 1 Introduction

Perimenopause and postmenopause are critical physiological transitions for women after the age of 40, accompanied by many physiological and psychological changes (Marnocha, Bergstrom, and Dempsey 2011). Perimenopause is the transitional period before women enter menopause (Brinton et al. 2015), while postmenopause is the stage in the female reproductive system when the menstrual cycle stops (Shuster et al. 2010). The typical characteristics of women in perimenopause and postmenopause are decreased ovarian function and endocrine disorders, which can cause psychological and physiological harm to women (Troìa et al. 2021). The most common symptoms during this period include vasomotor generalized hot flashes, menstrual changes, insomnia, dreaminess, and depression (Lialy et al. 2023; Tandon et al. 2022).

The long‐term persistence of perimenopausal and postmenopausal symptoms can lead to the occurrence of related diseases, especially metabolic‐related diseases, such as hypertension (Nash et al. 2003), coronary heart disease (Gast et al. 2011) and osteoporosis (Pérez et al. 2013). This is primarily because of the decreased estrogen levels in perimenopausal and postmenopausal women which can impact blood lipid metabolism, vascular function, and cardiovascular health. Estrogen plays an important role in maintaining the balance of lipid metabolism (Ko and Jung 2021; Ko and Kim 2020; Ryczkowska et al. 2023), and the decrease of estrogen levels in perimenopausal and postmenopausal women can result in increased levels of total cholesterol (TC), triglycerides (TGs), low‐density lipoprotein (LDL) and decreased levels of high‐density lipoprotein (HDL) (Han et al. 2024; Nie et al. 2022). Some studies have found that elevated TC and LDL lead to an increased risk of cardiovascular disease in postmenopausal women (Anagnostis et al. 2020; Gentile et al. 2020). Moreover, Postmenopausal osteoporosis is caused by declining estrogen levels, which leads to compromised bone strength and increased risk of fractures (Black and Rosen 2016). Osteocalcin is a special biochemical marker of bone turnover and bone formation, involved in bone mineralization and calcium homeostasis (Di Medio and Brandi 2021). A study has found that serum total osteocalcin is closely related to glucose and lipid metabolism in postmenopausal women and negatively correlated with TC, LDL, Fasting blood‐glucose (FPG), and Postprandial blood glucose (PBG).

The complex pathogenesis of cardiovascular disease and osteoporosis, long treatment cycles, and drug side effects have brought huge difficulties to their treatment (Barnsley et al. 2021; Pala et al. 2020). Estrogen has been used clinically to treat cardiovascular diseases and has achieved certain therapeutic effects (Shufelt and Manson 2021). And hormone replacement therapy (HRT) (Vigneswaran and Hamoda 2022) has been considered an effective treatment for postmenopausal osteoporosis and fractures. Several studies have shown HRT to be an effective treatment modality in treating cardiovascular disease (Grodstein et al. 1997) and osteoporosis (Cranney and Wells 2003; Gambacciani and Levancini 2014).

Although estrogen can be used to treat cardiovascular disease and bone disease in perimenopausal and postmenopausal women, HRT can cause some side effects, such as thrombosis and stroke (Genazzani et al. 2021; Khalil 2013). Studies have shown that HRT is more effective in treating postmenopausal women‐related disease, but patients need to pay attention to age and years since menopause (Schierbeck et al. 2012). In order to explore various physiological indicators of menopausal and perimenopausal women and solve the difficulties of HRT treatment, this study used the data of all perimenopausal and postmenopausal women aged 40–54 years old in West China Second University Hospital from October 2021 to October 2023. By detecting various physiological and biochemical indicators of women of different ages (perimenopausal and postmenopausal women), we can explore the impact of age on female menopause and the pathogenesis of perimenopausal women and postmenopausal women. This study provides insights into the pathogenesis of cardiovascular disease and bone disease in perimenopausal and postmenopausal women of different ages and provides new insights into HRT treatment of these diseases.

## 2 Methods

### 2.1 Study Participants

The subjects of this study were 1187 postmenopausal and perimenopausal females (Table S1) (Cheung et al. 2004) aged 40–54 years old registered at West China Second University Hospital in China from October 2021 to October 2023. Perimenopausal women (Tarlatzis and Zepiridis 2003) experience menstrual irregularities, decreased estrogen levels, and menopausal symptoms. Postmenopausal women (Belchetz 1994) are those who have experienced amenorrhea for more than 1 year. Women with one of the following conditions were excluded: (1) Other endocrine disorders, such as hypothyroidism and hypercortisolism. (2) Severe debilitating diseases, such as cancer, liver and kidney dysfunction. (3) Have the habit of smoking or drinking. (4) Have a history of estrogen replacement therapy. (5) People who cannot remember their last menstrual cycle. All participants in this study gave written informed consent, and this study was approved by West China Second Medical College of Sichuan University.

### 2.2 Data Collection of the Metabolic‐Related Diseases

Postmenopausal and perimenopausal women are often accompanied by various metabolic‐related diseases, such as cardiovascular system diseases (Atsma et al. 2006) and skeletal system diseases (Stevenson 2011). Among postmenopausal and perimenopausal women, the most common cardiovascular diseases are hypertension and coronary artery disease (Dosi et al. 2014). Participants with a systolic blood pressure greater than 140 mmHg or a diastolic blood pressure greater than 90 mmHg were considered hypertensive (Böhm et al. 2018). The common symptoms of coronary heart disease (chest pain, dyspnea, chest tightness, and easy fatigue) (Lu et al. 2015) and coronary angiography technology were combined to determine whether subjects had coronary heart disease. Dual‐energy X‐ray absorptiometry (DEXA) (Jain and Vokes 2017) is a diagnostic tool commonly (Di Carli and Hachamovitch 2007) used to assess bone density, especially to detect osteoporosis. According to World Health Organization (WHO) standards, a T‐score (T_1_) between −1 and − 2.5 is considered bone loss, while a T‐score (T_2_) below −2.5 is diagnosed as osteoporosis.

### 2.3 Measurements of Anthropometric Indexes

Subjects were barefoot and wearing light clothing, and their weight and height were measured. Body mass index (BMI) was calculated as the ratio of weight (kg) to height (m^2^) squared. Subjects' waist circumference (WC) was measured in an upright position between the 10th rib (lower costal arch) and the ilium (iliac crest). This location is typically at the most prominent point of the patient's abdomen, where the WC is the smallest. The subject's hip circumference (HC) was measured horizontally across the distance between the two upper ilium bones. Waist‐to‐hip ratio (WHR) is the ratio of WC to HC. Bioelectrical impedance measurement techniques (Kyle et al. 2004) were used to measure body fat ratio (BFR) in all subjects. DEXA was used to detect the bone mineral density (BMD) of all subjects, including the BMD and T_1_ value of the subjects’ lumbar vertebrae L2 to L4 (L2–L4), the BMD and T_2_ value of the subjects’ the femoral neck.

### 2.4 Laboratory Measurements

Medical staff used a syringe and needle to puncture the patient's vein to obtain a 10 mL blood sample from each fasting subject. After the fasting blood sample collection was completed, the same method was used to obtain a 10 mL blood sample from each subject within 120 min after all subjects took 75 g of glucose orally. Blood glucose concentrations were measured by electrochemical methods during fasting FPG and after oral glucose administration PBG in all subjects. Chemical analysis methods were used to detect TC, TG, HDL, and LDL levels in the blood of all subjects.

For menopausal women, we can use fasting blood samples from these subjects to measure blood sex hormones (follicle‐stimulating hormone (FSH), luteinizing hormone (LH), estradiol (E_2_), testosterone (T)) by enzyme‐linked immunosorbent assay (ELISA) (Butler 2000). For perimenopausal women, our sex hormone blood sample collection time is on the 3rd day of the menstrual cycle or after amenorrhea for 6 months or more than three cycles.

### 2.5 Statistical Analysis

SPSS 21.0 (Statistical Package for Social Sciences, Inc., Chicago, IL, USA) (George and Mallery 2019) and GraphPad prism5 (Statistical and Graphing Software, China) (Motulsky 2007) were used for data analysis and graphing. All normally distributed data in this study are expressed as mean ± standard deviation (SD), while data with skewed distribution are expressed as median (interquartile range). The chi‐square test was used to evaluate whether there were significant differences in the proportion of related diseases among the groups (McHugh 2013). Bivariate correlation analysis evaluates the effects of hormones and age on TC, LDL, T_1_ and T_2_ of the four indicators and then uses the correlation coefficient to detect the degree of association. All statistical tests in this study were performed at a significance level of 0.05, and Bonferroni correction (Weisstein 2004) was used for multiple comparisons.

## 3 Results

### 3.1 Subjects

This study obtained a total of 1187 postmenopausal and perimenopausal female cases from West China Second University Hospital, and a total of 414 female cases met the screening exclusion requirements. Finally, 773 female cases met the requirements of this study. Of the 773 female cases, 567 were perimenopausal, and 206 were postmenopausal. Among these 773 female cases, 78 had cardiovascular disease (hypertension and coronary heart disease), and 218 had bone loss and osteoporosis. This result showed that the prevalence of skeletal system diseases in postmenopausal and perimenopausal women is significantly higher than that of cardiovascular system diseases.

In order to explore the impact of age on menopausal and perimenopausal women, we divided menopausal and perimenopausal women aged 40–54 into three groups, namely 40–44 years (*n* = 314), 45–49 years (*n* = 305) and 50–54 years (*n* = 154) (Table 1). Of the 567 perimenopausal women, 280 were aged 40–44 years, 234 were aged 45–49 years, and 52 were aged 50–54 years. Among the 206 Postmenopausal women, 34 were aged 40–44, 71 were aged 45–49, and 102 were aged 50–54. The number of perimenopausal women decreases with age, while the number of menopausal women continues to increase. This also suggested that increasing age can lead to the transition from perimenopausal women to menopausal women.

**TABLE 1:** Probability of cardiovascular system diseases and skeletal system diseases among perimenopausal and postmenopausal women at different age stages.

Age	Number		Cardiovascular diseases		Skeletal system	
	Peri‐M	Post‐M	Peri‐M	Post‐M	Peri‐M	Post‐M
40–44	281	33	12 (4.27%)	0	54 (19.29%)	7 (21.21%)
45–49	234	71	25 (10.68%)	9 (12.68%)	62 (26.50%)	28 (39.44%)
50–54	52	102	10 (19.23%)	22 (21.57%)	16 (30.77%)	51 (50.00%)
Total	567	206	47 (8.29%)	31 (15.05%)	132 (23.28%)	86 (41.75%)

*Note:* Peri‐M means perimenopausal and Post‐M means postmenopausal.

### 3.2 Metabolic‐Related Diseases in Perimenopausal and Postmenopausal Women

**FIGURE 1:**
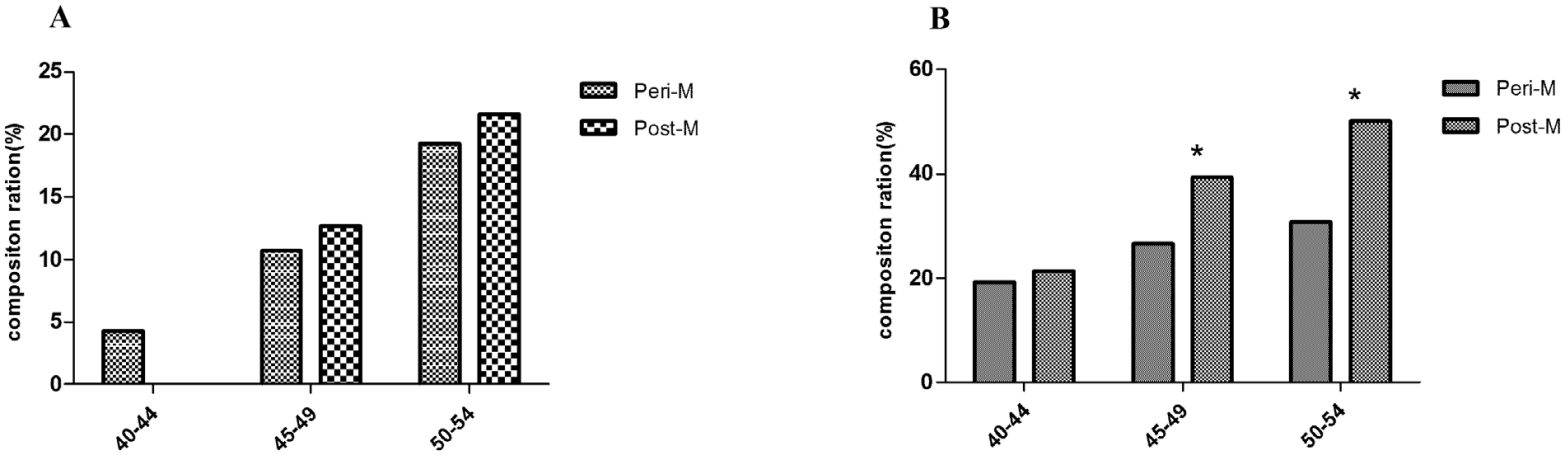
The probability of cardiovascular disease and skeletal system disease in the perimenopausal and postmenopausal women at different ages. (A) Cardiovascular disease. (B) Skeletal diseases. *Means *p* < 0.05, Peri‐M means perimenopausal, Post‐M means postmenopausal.

We analyzed the probability of cardiovascular system diseases and skeletal system diseases in perimenopausal and postmenopausal women at different ages (Figure 1). Among cardiovascular diseases, the overall prevalence probability of perimenopausal women was 8.29%, and the prevalence rates of perimenopausal women at 40–44, 45–49, and 50–54 were 4.29%, 10.68%, and 19.23% respectively. The total prevalence probability of cardiovascular disease in postmenopausal women was 15.15%, and the probability of cardiovascular disease in 40–44, 45–49, and 50–54 years were 0%, 12.68%, and 21.57%. Among skeletal system diseases, the overall prevalence probability of perimenopausal women was 23.28%, while the overall prevalence probability of postmenopausal women was 41.75%. In the 40–44, 45–49, and 50–54 age groups, the overall prevalence probability of perimenopausal women suffering from skeletal diseases were 19.29%, 26.50%, and 30.77%, respectively. The prevalence probabilities of postmenopausal women were 20.59%, 39.44%, and 50.00%. This result showed that the probability of perimenopausal and postmenopausal women suffering from cardiovascular disease and bone disease increases with age increases, and the probability of disease in postmenopausal women is higher than that of perimenopausal women.

Correlation analysis between cardiovascular disease and bone disease and perimenopausal and postmenopausal women analysis showed that there is no correlation between cardiovascular disease in perimenopausal and postmenopausal women. There was a significant difference between perimenopausal and postmenopausal women in the 45–49 and 50–54 age groups (*p* < 0.05), and the incidence probability of postmenopausal women was significantly higher than that of perimenopausal women.

### 3.3 Physiological and Biochemical Indicators of Perimenopausal and Postmenopausal women

In order to explore the influencing factors of perimenopausal and postmenopausal women, we analyzed the physiological indicators of perimenopausal and postmenopausal women (Table 2). The values of FPG, PBG, BMI, WC, HC, BFR, TG, and HDL in perimenopausal and postmenopausal women were consistent, and there was no significant difference between them (*p* > 0.05). Except for testosterone, significant differences were observed in the levels of various sex hormones between perimenopausal and postmenopausal women. The contents of E_2_, FSH, and LH in perimenopausal women are 50.60 pg/mL, 36 IU/L, and 20.85 IU/L, respectively. The contents of these three hormones in postmenopausal women are 16.40 pg/mL, 79.30 IU/L, and 37.45 IU/L. The sex hormone E_2_ of postmenopausal women is significantly higher than that of postmenopausal women (*p* < 0.001), and FSH and LH are significantly lower than that of postmenopausal women (*p* < 0.001). Among various blood lipid indicators, only the TC content was significantly different between perimenopausal and postmenopausal women (*p* < 0.001), with the contents being 4.79 and 5.12 mmol/L. All four BMD indicators were significantly different between perimenopausal and postmenopausal women (*p* < 0.001). The BMD and T_1_ values of L2–L4, BMD and T_2_ values of the neck of the femur and T_2_ in perimenopausal women were 1.12, −0.07, 0.90, and −0.23, respectively, while the values in postmenopausal women were 1.03, −0.86, 0.86, and −0.61 respectively. These results suggested that perimenopausal women and postmenopausal women differ in some physiological markers that may be associated with the development of various metabolic diseases in women.

**TABLE 2:** Physiological and biochemical indicators of perimenoptreatment and postmenopausal women.

	Peri‐M (*n* = 567) Mean ± SD	Post‐M (*n* = 206) Mean ± SD	*p*
E_2_ (pg/mL)	50.60 (21.80–111.80)	16.40 (11.80–30.53)	0.000*
T (ng/mL)	0.32 (0.22–0.44)	0.32 (0.19–0.40)	0.169
FSH (IU/L)	36 (9.10–70.50)	79.30 (56.55–97.90)	0.000*
LH (IU/L)	20.85 (5.28–40.70)	37.45 (27.30–46.55)	0.000*
TC (mmol/L)	4.79 ± 0.82	5.12 ± 0.73	0.000*
TG (mmol/L)	1.05 (0.82–1.53)	1.16 (0.88–1.56)	0.068
HDL (mmol/L)	1.64 ± 0.37	1.66 ± 0.38	0.554
LDL (mmol/L)	2.67 ± 0.74	2.91 ± 0.67	0.000*
FPG (mmol/L)	5.20 (4.90–5.50)	5.26 (4.97–5.60)	0.209
PBG (mmol/L)	6.66 (6.45–6.87)	6.60 (6.27–6.89)	0.899
BMI (kg/m^2^)	22.74 ± 2.68	22.37 ± 2.80	0.097
WC (cm)	75 (70–80)	74 (69–80)	0.680
HC (cm)	90 (86.50–94)	90 (86–93)	0.353
WHR	0.83 ± 0.05	0.83 ± 0.05	0.994
BFR	32.75 (29.9–35.4)	31.80 (29.13–35.75)	0.128
L_2_–L_4_ (BMD)	1.12 ± 0.14	1.03 ± 0.15	0.000*
T_1_	−0.07 ± 1.16	−0.86 ± 1.22	0.000*
Neck of femur (BMD)	0.90 ± 0.13	0.86 ± 0.11	0.000*
T_2_	−0.23 ± 1.05	−0.61 ± 0.95	0.000*

*Note:* Normally distributed data were expressed as means ± SD, skewed distribution were reported as median (interquartile range), ab is the difference between groups of perimenopausal women and postmenopausal women, * indicates a *p* value less than 0.001.

### 3.4 Physiological and Biochemical Indicators of Perimenopausal and Postmenopausal Women at Different Ages

We further analyzed the differences in various physiological and biochemical indicators among perimenopausal and postmenopausal women at different ages to explore the impact of age on women's metabolism‐related diseases (Table 3). The values of T, TG, HDL, FPG, BMI, HC, WC, WHR, and BFR in perimenopausal and postmenopausal women at different ages were consistent (*p* > 0.05), and there was no significant difference between them.

**TABLE 3:** Physiological and biochemical indicators of perimenopausal and postmenopausal women at different ages.

	40–44 (*n* = 314) Mean ± SD	45–49 (*n* = 305) Mean ± SD	50–54 (*n* = 154) Mean ± SD	*p*
E_2_ (pg/mL)	56.45 (25.30–117.23)	31.10 (14.10–86.90)	17.85 (11.80–35.55)	0.000
T (ng/mL)	0.32 (0.23–0.44)	0.32 (0.21–0.42)	0.33 (0.19–0.44)	0.783
FSH (IU/L)	28.50 (8.38–74.73)	47.55 (13.10–77.13)	74.65 (52.90–91.43)	0.000
LH (IU/L)	17.65 (4.50–40.28)	26.40 (8.50–42.50)	35.30 (26.00–46.10)	0.000
TC (mmol/L)	4.71 ± 0.80	4.95 ± 0.81	5.07 ± 0.74	0.000
TG (mmol/L)	0.99 (0.79–1.43)	1.12 (0.85–1.56)	1.23 (0.97–1.68)	0.026
HDL (mmol/L)	1.64 ± 0.37	1.66 ± 0.38	1.64 ± 0.35	0.646
LDL (mmol/L)	2.62 ± 0.75	2.79 ± 0.74	2.87 ± 0.62	0.000
FPG (mmol/L)	5.20 (4.90–5.45)	5.20 (4.96–5.60)	5.27 (4.90–5.57)	0.220
PBG (mmol/L)	6.54 (5.70–6.60)	6.72 (6.21–6.72)	6.92 (6.70–6.92)	0.000
BMI (kg/m^2^)	22.63 ± 2.67	22.67 ± 2.71	22.61 ± 2.65	0.978
WC (cm)	74 (69–79)	75 (70–80)	75 (70–80)	0.129
HC (cm)	90.15 ± 5.80	90.82 ± 6.03	90.75 ± 6.04	0.323
WHR	0.83 ± 0.05	0.83 ± 0.05	0.83 ± 0.05	0.743
BFR	32.65 (29.48–35.03)	33 (29.95–35.6)	32.80 (30.08–35.63)	0.300
L_2_–L_4_ (BMD)	1.13 ± 0.13	1.11 ± 0.16	1.03 ± 0.14	0.000
T_1_	−0.03 ± 1.06	−0.23 ± 1.36	−0.86 ± 1.14	0.000
Neck of femur (BMD)	0.91 ± 0.12	0.88 ± 0.12	0.87 ± 0.13	0.030
T_2_	−0.18 ± 1.03	−0.42 ± 1.01	−0.48 ± 1.01	0.029

*Note:* Normally distributed data were expressed as means ± SD, and skewed distribution were reported as median (interquartile range).

Among various sex hormone indexes, E_2_, FSH and LH showed significant differences in perimenopausal and postmenopausal women at different ages, and all of them increased with age (*p* < 0.001). These changes may be closely related to changes in ovarian function, changes in hormone regulatory mechanisms, and other physiological processes. There were also significant differences in the three blood lipid indicators TC (*p* < 0.001), TG (*p* < 0.05), and LDL (*p* < 0.001) in perimenopausal and postmenopausal women at different ages that showed an increasing trend with age, which may be related to women's metabolism at different ages. The values of L2–L4 (*p* < 0.001), T_1_ (*p* < 0.001), neck of femur (*p* < 0.05), and T_2_ (*p* < 0.05) are significant differences in perimenopausal and postmenopausal women at different ages, and the values of L2–L4, T_1_, Neck of femur decrease with age, and T_2_ increases with age.

### 3.5 Effects of Age and E_2_ on Perimenopausal and Postmenopausal Women‐Related Indicators

Estradiol is one of the most important female estrogens and plays an important role in various physiological regulation processes of women. To exclude the influence of the weight of perimenopausal and postmenopausal women on the results, we calculated the BMI values of perimenopausal and postmenopausal women of different ages. In the 40–44, 45–49, and 50–54 age groups, the BMI value of perimenopausal women were 22.48 ± 2.50, 22.77 ± 2.22, and 22.91 ± 2.13, the BMI value of postmenopausal women were 22.78 ± 2.83, 22.27 ± 3.28 and 22.44 ± 2.76. This result indicated that the results of this study can't be affected by the BMI values of the subjects. This result showed that there is no difference in BMI values in perimenopausal and postmenopausal women at different ages (*p* > 0.05).

**FIGURE 2:**
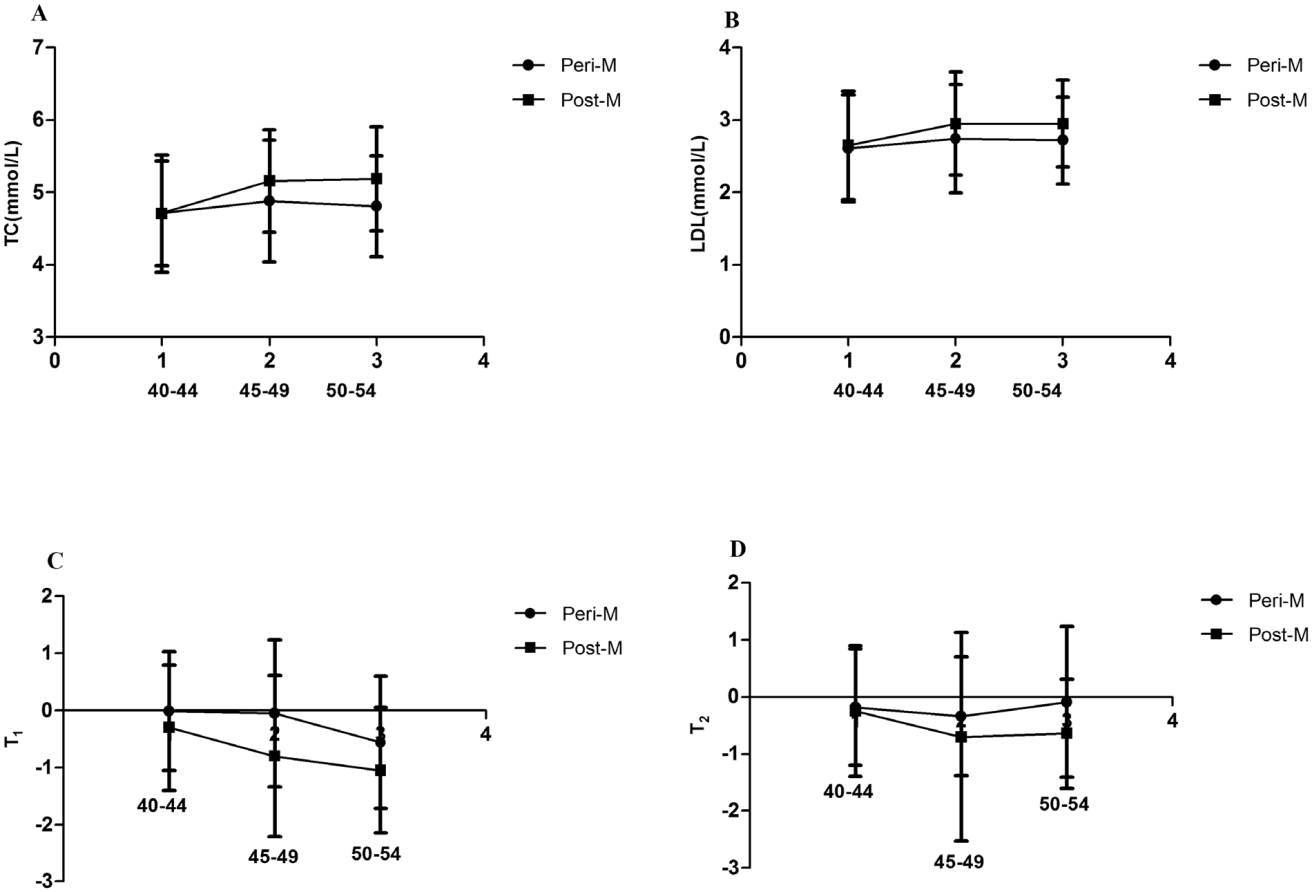
Distribution of four indicators among perimenopausal and postmenopausal women at different ages. (A) TC. (B) LDL. (C) T_1_. (D) T_2_. TC means total cholesterol, LDL means low‐density lipoprotein, T_1_ means the subjects' lumbar vertebrae L2 to L4 (L2–L4), T_2_ means the subjects' the femoral neck.

Combining the results of various physiological and biochemical indicators in perimenopausal and postmenopausal women at different ages, we selected four indicators for further analysis to explore their correlation with sex hormone E_2_ and age, TC, LDL, T_1_, and T_2_. The four indicators remain consistent in perimenopausal women and postmenopausal women in the 40–44 stage (Figure 2). However, the TC, LDL, T_1_, and T_2_ values of postmenopausal women are higher than those of postmenopausal women with age increases. This study further analyzed the correlation between E_2_ and four indicators, and the correlation between age and four indicators. The results showed that E_2_ and age were significantly related to four indicators among perimenopausal women and menopausal women (*p* < 0.001) (Table S2). The correlation coefficient showed that the correlation coefficient between age and the four indicators in perimenopausal women and menopausal women is higher than that between E_2_ and the four indicators (Table 4). This result suggested that age plays a key role in the development of metabolic diseases in perimenopausal and postmenopausal women.

**TABLE 4:** The differences of correlation in the E2 and age with four indicators.

*r*	TC		LDL		T_1_		T_2_	
	Peri‐M	Post‐M	Peri‐M	Post‐M	Peri‐M	Post‐M	Peri‐M	Post‐M
E_2_	0.048485	−0.09646	−0.0033	−0.06326	0.043969	0.118806	0.037959	0.124804
Age	0.530844	0.207316	0.088003	0.185472	−0.20632	−0.18263	−0.03327	−0.04261

## 4 Discussion

This study analyzed estrogen and blood lipid levels, changes in body indicators, and metabolic‐related diseases in 773 perimenopausal and postmenopausal women. Postmenopausal women have higher rates of cardiovascular disease and bone disease than perimenopausal women. Moreover, estrogen and BMD decrease, and TC and LDL levels increase in perimenopausal and postmenopausal women with age increases.

### 4.1 Cardiovascular Disease

The likelihood of developing cardiovascular disease gradually rises due with age to aging‐related physiological changes, such as vascular stiffening, increased arterial plaque buildup, and a decline in metabolic function (Newman et al. 2003). Moreover, dyslipidemia is a common risk factor for cardiovascular disease, mainly manifested in increased TC, TG, and LDL, decreased HDL levels (Gentile et al. 2020). In the results of this study, the TC and LDL levels of postmenopausal women increased significantly, and the TC and LDL levels of women can further increase with the increase of women's age. This suggested that estrogen deficiency with age is a key factor in the increased risk of cardiovascular disease after menopause. During perimenopause and menopause, women's estrogen levels decreased, which can increase the risk of cardiovascular disease (Gentile et al. 2020). Postmenopausal women had a significantly increased risk of cardiovascular disease, particularly the incidence of myocardial infarction and coronary heart disease (Bertoia et al. 2012). In addition, perimenopausal and postmenopausal women may also be affected by other cardiovascular risk factors, such as dyslipidemia, hypertension, and obesity (Hulley et al. 1998).

Estrogen can promote the secretion of bile acids in the liver, which can lead to an increase in cholesterol excretion in the liver and a decrease in cholesterol levels in the body (Lavoie 2016). It can also protect blood vessels by improving plasma lipid profiles and lipid peroxidation (Wang et al. 2009) and participate in the pathophysiological responses of blood vessels through genetic or non‐genetic regulation of estrogen receptor‐mediated expression of vascular endothelial cells and smooth muscle cells (Phelps et al. 2019). The menopausal transition is a complex physiological stage in which a woman's body undergoes dramatic changes in hormone levels, including decreases in estrogen and LH levels. Changes in these hormones can impact lipid metabolism and the stability of blood lipid profiles that, lead to alterations in HDL levels (Duntas and Brenta 2018). Some studies have shown that estrogen has an effect on HDL synthesis and metabolism, and its reduction may be related to reduced estrogen levels (Palmisano, Zhu, and Stafford 2017). Additionally, changes in body fat distribution, metabolic rate and other factors may also influence variations in HDL levels. (Després 2012).

### 4.2 Skeletal System Disorders

Estrogen plays an important role in the skeletal system (Turner, Riggs, and Spelsberg 1994). Its effects are mainly reflected in inhibiting the formation, activity, and lifespan of osteoclasts and increasing the recruitment, proliferation, differentiation and lifespan of osteoblasts (Cheng, Chen, and Chen 2022; Uehara, Soldi, and Silva 2020). In our study, we found that postmenopausal women had higher rates of bone loss and osteoporosis than perimenopausal women in any age group. This suggested that estrogen deficiency during menopause is one of the major causes of bone loss. We observed a decrease in BMD at the lumbar spine and femoral neck with age but not with menopausal status. The lumbar spine responded more sensitively to estrogen deficiency and loses density more rapidly. There was a nonlinear relationship between lumbar spine and femoral neck density and estrogen levels during perimenopause and a linear relationship after menopause. This suggested that menopause is a turning point in rapid changes in bone density, with bone density gradually declining as estrogen levels decrease. Decreased bone density is a common phenomenon in women during postmenopause (Lakshmanan et al. 2021). The lumbar spine and femoral neck are the most susceptible areas and their bone density gradually decreases with menopause. In particular, the lumbar spine is more sensitive to changes in estrogen levels, and bone density declines faster (Shieh et al. 2021). The main reason for the decrease in bone density in women during menopause is the decrease in estrogen levels, which causes the skeletal system to lose its protective effect. Additionally, women in early menopause have a higher risk of osteoporosis and fractures than women who go through menopause later in life.

BMI has a certain impact on osteoporosis in menopausal and perimenopausal women (Kanto et al. 2022). Studies have found that women with lower body weight are more likely to have a higher risk of osteoporosis (Shieh et al. 2022). During perimenopause and menopause, significant changes in female hormone levels are seen. The decline accelerates the loss of bone density, and women with a low BMI may be at greater risk of osteoporosis after menopause. A study of 68 anorexic patients and 30 healthy controls found that young anorexic women had BMD significantly below the normal range and that lean body mass had a significant effect on BMD (Villa et al. 2024). Moreover, patients with anorexia are at higher risk of osteoporosis in the lumbar spine and femoral regions, and sarcopenia is also an important influencing factor. Another study found BMD and hip bone strength parameters in obese sarcopenic women by comparing obese premenopausal women with sarcopenia and normal appendicular lean mass (ALM)/ BMI ratios (Hammoud et al. 2020). It was significantly lower than obese women with a normal ALM/BMI ratio, indicating that muscle mass has an important impact on bone health. In addition to BMI and age, the incidence of osteoporosis in women is also affected by education level, number of childbearing years, history of hypertension and diabetes, drinking history, age at menarche, age at menopause, and the use of estrogen and vitamin D (Long et al. 2023).

## 5 Conclusion

Compared with normal women, perimenopausal and postmenopausal women have an increased risk of metabolic‐related diseases, especially cardiovascular disease and osteoporosis. In this study, we found that the incidence of cardiovascular disease and osteoporosis is higher in menopausal women than in perimenopausal women and that the incidence of osteoporosis is associated with age in menopausal women. Moreover, estrogen and bone density decreased significantly compared with normal women in perimenopausal and postmenopausal women, while TC and LDL levels increased. Furthermore, we found that estrogen E_2_ was significantly associated with TC, LDL, and bone density. This study provides important insights into understanding the physiological and biochemical changes in perimenopausal and menopausal women and the mechanisms of related metabolic diseases that can provide new directions and ideas for future clinical research and clinical practice.

## Author Contributions

**Xiaoyan Luo:** formal analysis (equal), software (equal), supervision (equal), writing – original draft (lead). **Jun Zhang:** investigation (equal), methodology (equal), supervision (equal). **Yichuan Guo:** supervision (equal). **Liangzhi Xu:** conceptualization (lead), resources (lead), writing – review and editing (lead).

## Ethics Statement

The study protocol was approved by the medical ethics committee of West China Second University Hospital, Sichuan University in accordance with ethical guidelines.

## Consent

This study was approved by West China Second Medical College of Sichuan University.

## Conflicts of Interest

The authors declare no conflicts of interest.

## Supporting information


**Table S1** Original data of 773 perimenopausal and postmenopausal women.

**Table S2** The *p* value in the E_2_ and age with four indicators.

## Data Availability

The raw data during the current study are available in Table S1.
