# Supplementary material for: Association of Changes in Relevant Indicators With Cardiovascular Disease and Osteoporosis in Perimenopausal and Postmenopausal Women
Source: Food Sci Nutr. 2024 Nov 6;12(12):10296–305. doi: 10.1002/fsn3.4512 (PMC11666984; doi:10.1002/fsn3.4512)
Supplement: Supplementary file 2 — Table S2 The p value in the E2 and age with four indicators. [file FSN3-12-10296-s001.docx]

Table S2 The *p* value in the E_2_ and age with four indicators

| *p* | TC | | LDL | | T_1_ | | T_2_ | |
| --- | --- | --- | --- | --- | --- | --- | --- | --- |
|  | Peri-M | Post-M | Peri-M | Post-M | Peri-M | Post-M | Peri-M | Post-M |
| E2 | *** | *** | *** | *** | *** | *** | *** | *** |
| Age | *** | *** | *** | *** | *** | *** | *** | *** |

Note: *** means *p* < 0.001
